# Supplementary material for: The impact of chronic pain on brain gene expression
Source: medRxiv. 2024 May 21:2024.05.20.24307630. Preprint. [Version 1] doi: 10.1101/2024.05.20.24307630 (PMC11142271; doi:10.1101/2024.05.20.24307630)
Supplement: Supplement 1 [file media-1.pdf]

Cell-type deconvolution with an alternate reference dataset

We repeated cell type deconvolution of expression data from our bulk tissue samples with CIBERSORTx [1], using an alternate reference dataset downloaded from Gene Expression Omnibus[2] (Series GSE195445) and associated with a set of RNA-seq experiments using amygdala samples only [3]. First the Yu et al human dataset Seurat[4] object was processed to produce the files required for CIBERSORTx web application signature matrix generation step (columns of samples and rows of genes, with expression values expressed in TPM). Conversion to TPM using DGEobj.utils function 'convertCounts' requires gene lengths, which were obtained using biomaRt[5] and querying using gene symbols ('external_gene_name') to find start and end positions of each gene. This produced a reference expression dataset for 15, 387 genes from three human donors, each with seven cell types tested (astrocytes, endothelial cells, excitatory neurons, inhibitory neurons, microglia, oligodendrocytes, and oligodendrocyte progenitor cells). Then we performed CIBERSORTx step 2, using the generated signature matrix and Girgenti et al postmortem bulk tissue expression data in TPM. We then calculated the mean cell type proportion for each brain region, and compared this with cell type proportion means using our previous reference data set as described in the main manuscript.

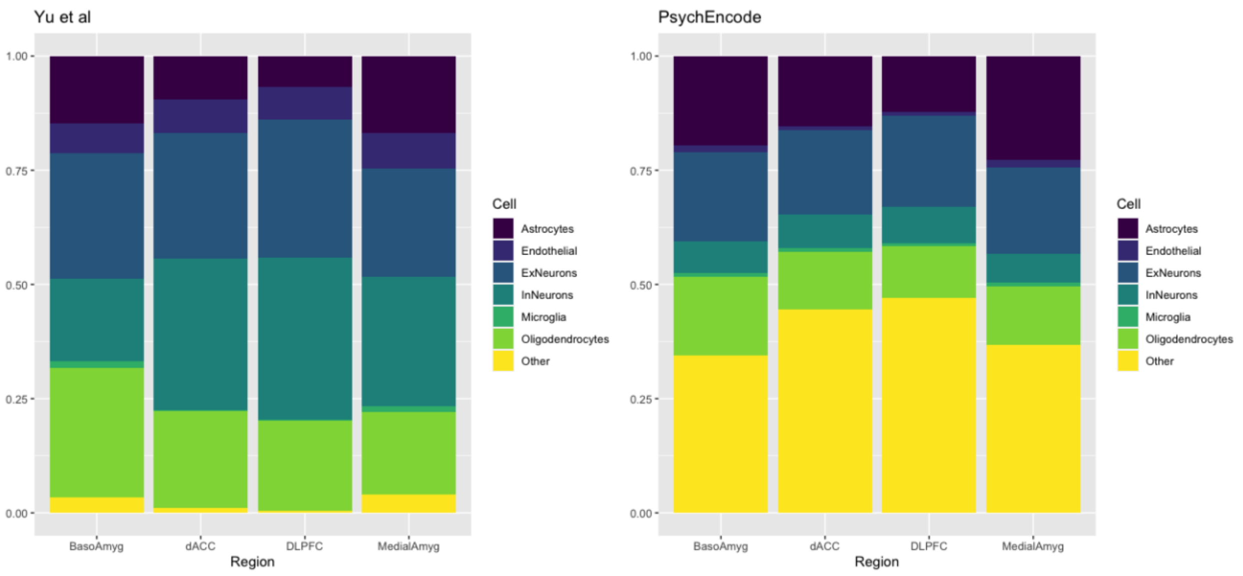

Cell type proportions across the four brain regions following cell-type expression imputation using amygdala (Yu et al) and cortex (psychENCODE) reference panels. Note: 'Other' in Yu et al is OPC, in PsychEncode 'Neurons'.

Significant differences in cell type proportions are seen, particularly in overall neuron content. We opted to impute cell type level expression data in cortical and amygdala bulk data separately using the two distinct reference data sets (psychENCODE cortical samples for dACC and DLPFC, and Yu et al amygdala samples for MeA and BLA).

Additional DEG analysis multiple-testing correction

We consider the five separate sets (PRS, oxymorphone, fentanyl, chronic pain, and migraine) of DEG analyses not to require additional cross-experiment multiple testing correction, i.e., multiple testing correction as described in the main manuscript is sufficient. However, we applied an additional, even more stringent DEG regression p value threshold (0.05 / number of genes tested within that tissue and cell type / 5 (number of traits in total across entire study)). While this does reduce the number of significant DEGs identified, a t-test comparing N DEGs at the original p value threshold vs. additional correction threshold p = 0.49, and in particular the main bulk of our chronic pain findings remain in the BLA microglia (original p value threshold N DEG in BLA microglia: 1810 (77% of all chronic pain DEGs), with additional correction: 1260 (82%)).

FUMA gene-set enrichment p value threshold

In order to calculate a Bonferroni-adjusted p value threshold we downloaded the entirety of the gene sets used in the latest version of FUMA GENE2FUNC from the FUMA website 'GENE2FUNC_genesets_v156plus.tar.gz'. We then removed duplicated gene sets and sets that were nested (gene sets for FUMA are sourced from MSigDB [6] and all are included despite e.g., certain sets being repeated due to their classification as both a canonical pathway and a reactome pathway (child category of canonical pathway)). This results in a total of 32, 584 independent gene sets and therefore a Bonferroni p value threshold of 0.05/32,584 within each tissue-cell type gene-set enrichment (pathway) analysis.

DEGs in Oligodendrocyte Progenitor Cells (OPCs)

We find 18 significant ($P_{Bonferroni} < 0.05$) chronic pain DEGs, 47 fentanyl DEGs, 1 oxymorphone DEG, 3 migraine DEGs, and 0 PRS-DEGs in MeA OPCs. We find 4 significant chronic pain DEGs, 64 fentanyl DEGs, 3 oxymorphone DEGs, 0 migraine DEGs, and 0 PRS-DEGs in BLA OPCs.

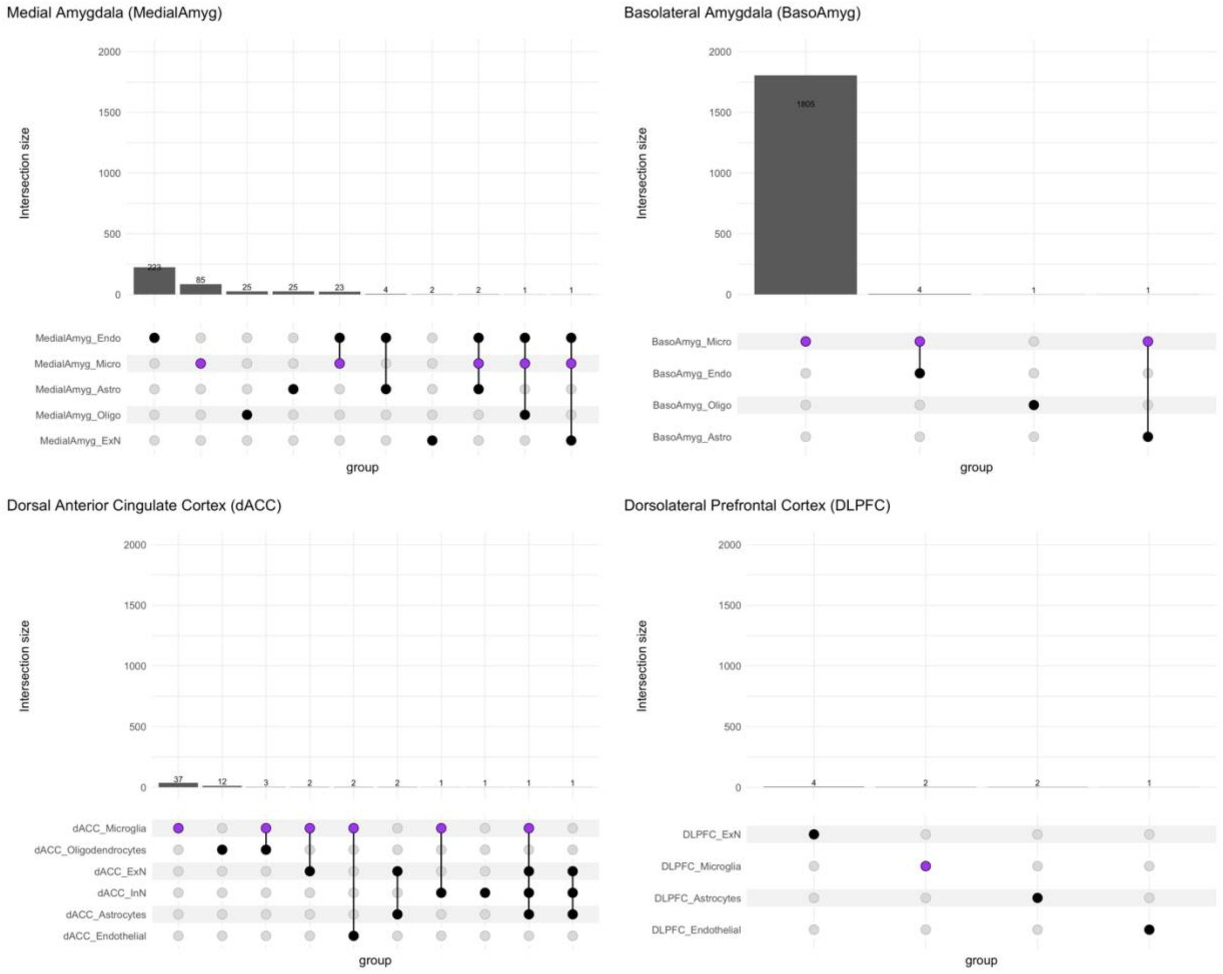

Fig S1: Cell type DEGs in chronic pain – comparison across regions and cell types. Majority of findings are in BLA microglia, and number of DEGs shared between cell types is low.

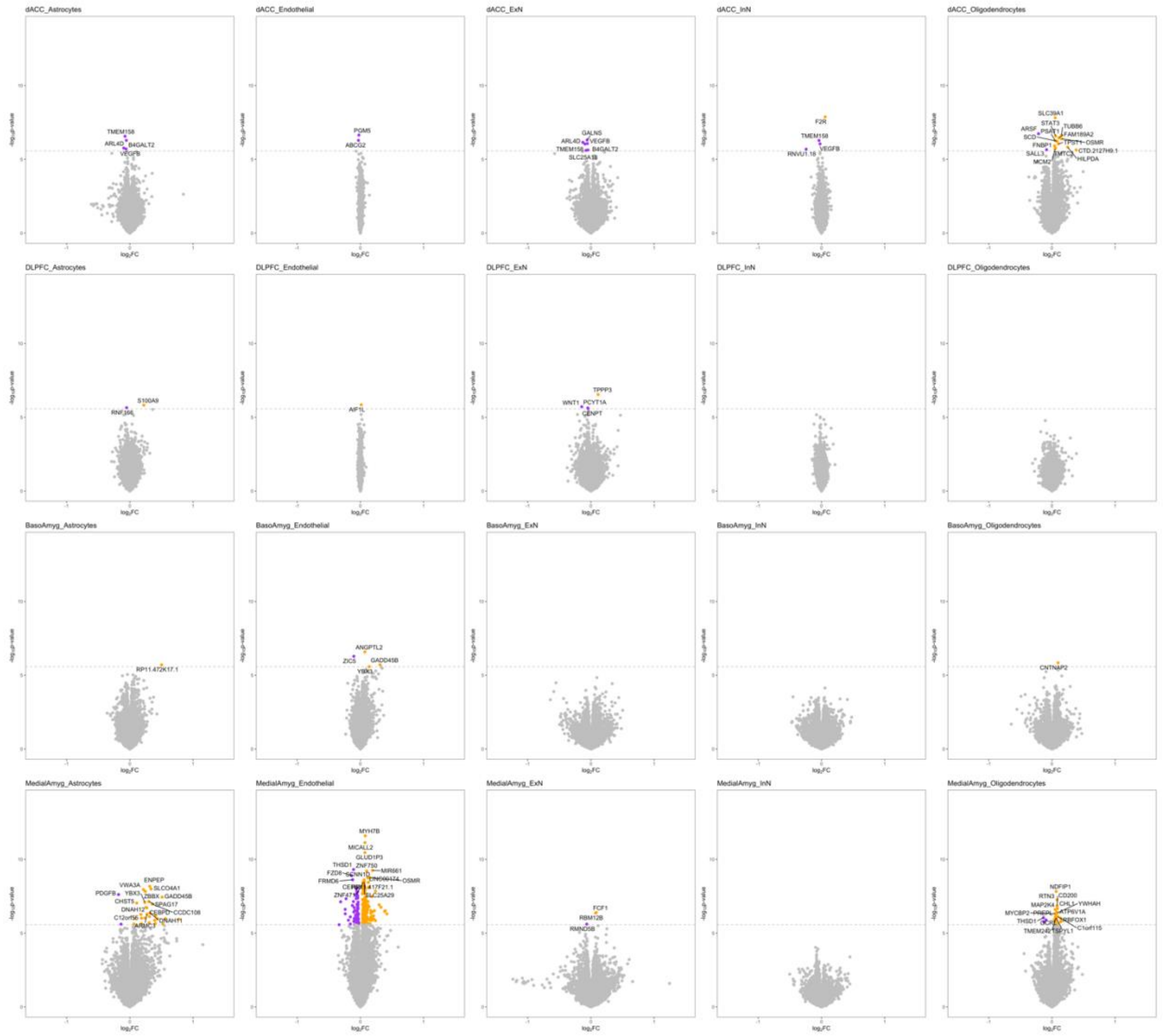

Fig S2: Additional genes differentially expressed across cell types in chronic pain. Chronic pain cell type DEGs in cell types per region. Purple = significantly ($P_{Bonferroni} < 0.05$) downregulated, orange = significantly ($P_{Bonferroni} < 0.05$) upregulated, dotted line = p value significance threshold. For legibility only the top 15 DEGs are labeled. FC = fold change. InN = inhibitory neuron, ExN = excitatory neuron.

Table S1: DEG analyses per-gene regression models. BLA = Basolateral Amygdala, MeA = Medial Amygdala, InN = inhibitory neuron, ExN = excitatory neuron, dACC = dorsal anterior cingulate cortex, DLPFC = dorsolateral prefrontal cortex, SV = surrogate variable, model = DEG regression model fitted per gene. In model column; 'gene' = gene expression value, 'trait' = trait value (case/control, yes/no for oxymorphone lifetime use, fentanyl lifetime use, migraine, chronic pain, quantitative MCP-PRS value for PRS).

Trait	Analysis type	Region	Cell	N SVs	model
Chronic Pain	Bulk	DLPFC	NA	22	gene ~ trait + SVs + Handedness + Migraine + Amphetamines
		dACC	NA	21	gene ~ trait + SVs + Handedness + Tramadol + Amphetamines + Ketamine + Anticholinergics..Antiparkinsonians + personal_count
		BLA	NA	25	gene ~ trait + SVs + Manner_of_death_Suicide + Delta.9.THC.Active + Barbiturates + witness_any
		MeA	NA	27	gene ~ trait + SVs + PTSD_PrimaryDx + Barbiturates + personal_count
	Cell-type	BLA	InN	53	gene ~ trait + SVs + Acetone + Cocaethylene + Benzoylecgonine_mg_L_blood + Hydrocodone + Ketamine + Anticholinergics..Antiparkinsonians + Hallucinogens + ECT
			ExN	43	gene ~ trait + SVs + Isopropanol + Famhx.Suicide + combat_only_count
			Oligodendrocytes	44	gene ~ trait + SVs + X11.Hydroxy.Delta.9.THC.Active + Amphetamines + Barbiturates + Ketamine + personal_any
			Endothelial	38	gene ~ trait + SVs + Race + BMI..calculated. + X11.Hydroxy.Delta.9.THC.Active + Barbiturates + disaster_count + assault_count
			Astrocytes	49	gene ~ trait + SVs + Isopropanol + X6.AM + Tramadol +

					Anticholinergics..Antiparkinsonians + Hallucinogens + EA_UA_neglect_count + personal_count
			Microglia	28	gene ~ trait + SVs + Handedness + BMI..calculated. + Isopropanol + X11.Hydroxy.Delta.9.THC.Active + Benzoylecgonine + Barbiturates + Ketamine + Anticholinergics..Antiparkinsonians + Hallucinogens + SA_count + witness_any
		dACC	InN	39	gene ~ trait + SVs
			ExN	40	gene ~ trait + SVs + Cocaethylene + Amphetamines + childhood_count
			Oligodendrocytes	55	gene ~ trait + SVs + Cocaethylene + Benzoylecgonine_mg_L_blood + Nicotine_ng_mL + Barbiturates + Anticholinergics..Antiparkinsonians
			Endothelial	21	gene ~ trait + SVs + Cocaethylene + Nicotine_ng_mL + Amphetamines + Barbiturates + disaster_count + childhood_count
			Astrocytes	43	gene ~ trait + SVs + Cocaethylene + Amphetamines + assault_count
			Microglia	12	gene ~ trait + SVs + Handedness + Acetone + Ethanol_mg_dL + X11.Hydroxy.Delta.9.THC.Active + Benzoylecgonine + Hydrocodone + Barbiturates + Anticholinergics..Antiparkinsonians + Past.Self.Mutilation
		DLPFC	InN	38	gene ~ trait + SVs + BMI..calculated. + Cocaethylene + Hallucinogens + witness_count + personal_count
			ExN	37	gene ~ trait + SVs + Cocaethylene + Barbiturates + Hallucinogens + witness_count
			Oligodendrocytes	54	gene ~ trait + SVs + Handedness +

					Barbiturates + Ketamine + Anticholinergics..Antiparkinsonians + ECT + witness_count + personal_count + combat_occ_count
			Endothelial	17	gene ~ trait + SVs + Manner_of_death_Accident + GERD + X11.Hydroxy.Delta.9.THC.Active + Benzoylecgonine_mg_L_blood + Barbiturates + Delusions + combat_occ_count
			Astrocytes	39	gene ~ trait + SVs + X11.Hydroxy.Delta.9.THC.Active + Amphetamines + personal_count + combat_occ_count
			Microglia	8	gene ~ trait + SVs + Handedness + Manner_of_death_Accident + Isopropanol + X11.Hydroxy.Delta.9.THC.Active + Benzoylecgonine_mg_L_blood + Anticholinergics..Antiparkinsonians + Anti.Inflammatories + Delusions + combat_occ_count
		MeA	InN	43	gene ~ trait + SVs + Acetone + X11.Hydroxy.Delta.9.THC.Active + Barbiturates + Ketamine + Hallucinogens + disaster_count + childhood_count
			ExN	50	gene ~trait + SVs + Manner_of_death_Suicide + Isopropanol + Cocaethylene + Ketamine + Anticholinergics..Antiparkinsonians + ECT
			Oligodendrocytes	46	gene ~ trait + SVs + GERD + Acetone + X11.Hydroxy.Delta.9.THC.Active + Benzoylecgonine_mg_L_blood + Barbiturates + Ketamine + Anticholinergics..Antiparkinsonians + witness_count + combat_occ_count
			Endothelial	42	gene ~ trait + SVs + Nicotine_ng_mL +

					Barbiturates + Hallucinogens + disaster_count
			Astrocytes	51	gene ~ trait + SVs + Cocaethylene + Hallucinogens + Other.Drugs + ECT + witness_count
			Microglia	24	gene ~ trait + SVs + Acetone + Cocaethylene + Benzoylecgonine_mg_L_blood + Barbiturates + Ketamine + Anticholinergics..Antiparkinsonians + Hallucinogens + witness_count
Fentanyl	Bulk	DLPFC	NA	22	gene ~ trait + SVs + Handedness + Amphetamines + Barbiturates + personal_count + combat_occ_count
		dACC	NA	21	gene ~ trait + SVs + Handedness + Delta.9.THC.Active + Tramadol + Amphetamines + Ketamine + personal_count
		BLA	NA	25	gene ~ trait + SVs + Delta.9.THC.Active + Barbiturates + Anticholinergics..Antiparkinsonians + witness_any
		MeA	NA	27	gene ~ trait + SVs + PTSD_PrimaryDx + Hydrocodone + Barbiturates + personal_count
	Cell-type	BLA	InN	54	gene ~ trait + SVs + Acetone + X11.Hydroxy.Delta.9.THC.Active + Cocaethylene + Hydrocodone + Ketamine + Anticholinergics..Antiparkinsonians + Antipsychotics + Hallucinogens + ECT + disaster_count
			ExN	43	Gene ~ trait + SVs + Isopropanol + cocaethylene + witness_count + combat_occ_any
			Oligodendrocytes	43	gene ~ trait + SVs + X11.Hydroxy.Delta.9.THC.Active + Amphetamines + Barbiturates + Ketamine + disaster_count

			Endothelial	38	gene ~ trait + Svs + Handedness + X11.Hydroxy.Delta.9.THC.Active + Barbiturates + Hallucinogens + witness_count + personal_count
			Astrocytes	49	gene ~ trait + SVs + Ketamine + Anticholinergics..Antiparkinsonians + Hallucinogens + ECT + witness_count + personal_count
			Microglia	27	gene ~ trait + SVs + Isopropanol + X11.Hydroxy.Delta.9.THC.Active + Cocaethylene + Barbiturates + Ketamine + Anticholinergics..Antiparkinsonians + Hallucinogens + ECT + witness_count + personal_count
		dACC	InN	38	gene ~ trait + SVs + Isopropanol + Anti.Inflammatories + personal_count
			ExN	40	gene ~ trait + SVs + Cocaethylene + Tramadol + personal_count
			Oligodendrocytes	55	gene ~ trait + SVs + Cocaethylene + Benzoylecgonine_mg_L_blood + Hydrocodone + Barbiturates + Anticholinergics..Antiparkinsonians + witness_count
			Endothelial	21	gene ~ trait + SVs + Cocaethylene + Nicotine_ng_mL + Amphetamines + Barbiturates + disaster_any + childhood_count
			Astrocytes	43	gene ~ trait + SVs + Cocaethylene + Amphetamines + Past.Self.Mutilation + Famhx.Suicide + combat_occ_any
			Microglia	13	gene ~ trait + SVs + Handedness + Acetone + Ethanol_mg_dL + X11.Hydroxy.Delta.9.THC.Active + Morphine + Anticholinergics..Antiparkinsonians + witness_count

		DLPFC	InN	38	gene ~ trait + SVs + Cocaethylene + Hallucinogens + witness_count + personal_count
			ExN	36	gene ~ trait + SVs + Cocaethylene + Benzoylecgonine_mg_L_blood + Barbiturates + Hallucinogens + witness_count
			Oligodendrocytes	53	gene ~ trait + SVs + Barbiturates + Ketamine + Anticholinergics..Antiparkinsonians + ECT + witness_count + personal_count + combat_occ_count
			Endothelial	17	gene ~ trait + SVs + GERD + X11.Hydroxy.Delta.9.THC.Active + Cocaethylene + Barbiturates + ECT + witness_count + personal_count + combat_occ_count
			Astrocytes	38	gene ~ trait + SVs + Cocaethylene + Amphetamines + childhood_count
			Microglia	8	gene ~ trait + SVs + Manner_of_death_Accident + Ethanol + Isopropanol + X11.Hydroxy.Delta.9.THC.Active + Anti.Inflammatories + EA_UA_neglect_count + personal_count + combat_only_count
		MeA	InN	44	gene ~ trait + SVs + Acetone + Barbiturates + Ketamine + Anticholinergics..Antiparkinsonians + Hallucinogens + ECT + disaster_count
			ExN	50	gene ~ trait + SVs + Isopropanol + Barbiturates + Ketamine + Anticholinergics..Antiparkinsonians + Hallucinogens + EA_UA_neglect_count
			Oligodendrocytes	45	gene ~ trait + SVs + Acetone + X11.Hydroxy.Delta.9.THC.Active + Benzoylecgonine +

					Amphetamines + Barbiturates + Ketamine Anticholinergics..Antiparkinsonians witness_count
			Endothelial	41	gene ~ trait + SVs + Nicotine_ng_mL + Barbiturates + Hallucinogens + disaster_count + childhood_count
			Astrocytes	50	gene ~ trait + SVs + Cocaethylene + Benzoylecgonine_mg_L_blood + Hallucinogens + ECT + witness_count
			Microglia	24	gene ~ trait + SVs + Handedness + Isopropanol + Barbiturates + Ketamine + Anticholinergics..Antiparkinsonians + Hallucinogens + witness_count + personal_count
Oxymorphone	Bulk	DLPFC	NA	22	gene ~ trait + SVs + Amphetamines + personal_count + combat_occ_count
		dACC		21	gene ~ trait + SVs + Delta.9.THC.Active + Amphetamines + Ketamine + personal_any
		BLA		25	gene ~ trait + SVs + Delta.9.THC.Active + Anticholinergics..Antiparkinsonians + witness_count
		MeA		27	gene ~ trait + SVs + Barbiturates + Hx.Other.Trauma +
	Cell-type	BLA	InN	54	gene ~ trait + SVs + Acetone + Delta.9.Carboxy.THC.Inactive + Ketamine + Anticholinergics..Antiparkinsonians + Hallucinogens + ECT + disaster_count
			ExN	42	gene ~ trait + SVs + Isopropanol + Cocaethylene + Famhx.Suicide + combat_only_count
			Oligodendrocytes	44	gene ~ trait + SVs + Amphetamines + Barbiturates + Ketamine + EA_UA_neglect_count + disaster_count

			Endothelial	38	gene ~ trait + SVs + Handedness + X11.Hydroxy.Delta.9.THC.Active + Barbiturates + Hallucinogens + witness_count + personal_count
			Astrocytes	49	gene ~ trait + SVs + Isopropanol + Ketamine + Anticholinergics..Antiparkinsonians + Hallucinogens + ECT + witness_count + personal_count
			Microglia	28	gene ~ trait + SVs + Handedness + Isopropanol + X11.Hydroxy.Delta.9.THC.Active + Barbiturates + Ketamine + Anticholinergics..Antiparkinsonians + Hallucinogens + ECT + witness_count + personal_count
		dACC	InN	39	gene ~ trait + SVs + Isopropanol + Benzoylecgonine_mg_L_blood + Anti.Inflammatories
			ExN	40	gene ~ trait + SVs + Cocaethylene + Amphetamines + personal_count
			Oligodendrocytes	55	gene ~ trait + SVs + Cocaethylene + Benzoylecgonine_mg_L_blood + Barbiturates + Anticholinergics..Antiparkinsonians + witness_count
			Endothelial	20	gene ~ trait + SVs + Isopropanol + Cocaethylene + Nicotine_ng_mL + Amphetamines + Barbiturates + disaster_any + childhood_count
			Astrocytes	43	gene ~ trait + SVs + Cocaethylene + Benzoylecgonine_mg_L_blood + witness_count
			Microglia	12	gene ~ trait + SVs + Handedness + Acetone + Ethanol_mg_dL + X11.Hydroxy.Delta.9.THC.Active + Benzoylecgonine + Hydrocodone + Barbiturates +

					Anticholinergics..Antiparkinsonians
		DLPFC	InN	38	gene ~ trait + SVs + BMI..calculated. + Cocaethylene + Hallucinogens + witness_count + personal_count
			ExN	37	gene ~ trait + SVs + Delta.9.THC.Active + Benzoylecgonine_mg_L_blood + Hallucinogens + witness_count
			Oligodendrocytes	54	gene ~ trait + SVs + Handedness + Barbiturates + Anticholinergics..Antiparkinsonians + witness_count + personal_count + combat_occ_count
			Endothelial	17	gene ~ trait + SVs + Manner_of_death_Accident + Isopropanol + X11.Hydroxy.Delta.9.THC.Active + Amphetamines + Barbiturates + Delusions + personal_count + combat_occ_count
			Astrocytes	38	gene ~ trait + SVs + Amphetamines + personal_count
			Microglia	8	gene ~ trait + SVs + Handedness + Manner_of_death_Accident + Isopropanol + X11.Hydroxy.Delta.9.THC.Active + Anti.Inflammatories + Delusions + personal_count + combat_occ_count
		MeA	InN	43	gene ~ trait + SVs + Acetone + Barbiturates + Ketamine + Hallucinogens + disaster_count + childhood_count
			ExN	49	gene ~ trait + SVs + Isopropanol + Barbiturates + Ketamine + Anticholinergics..Antiparkinsonians + EA_UA_neglect_count
			Oligodendrocytes	46	gene ~ trait + SVs + Other.Opiates + Barbiturates + Ketamine + Anticholinergics..Antiparkinsonians +

					witness_count + personal_count
			Endothelial	41	gene ~ trait + SVs + Handedness + X11.Hydroxy.Delta.9.THC.Active + Barbiturates + Anti.Inflammatories + witness_count + personal_count + combat_occ_count
			Astrocytes	51	gene ~ trait + SVs + Cocaethylene + Benzoylecgonine_mg_L_blood + Hallucinogens + ECT + witness_count
			Microglia	24	gene ~ trait + SVs + Acetone + Cocaethylene + Nicotine_ng_mL + Barbiturates + Ketamine + Anticholinergics..Antiparkinsonians + Hallucinogens + non_combat_count
Migraine	Bulk	DLPFC	NA	22	gene ~ trait + SVs + Handedness + Amphetamines + personal_count + combat_occ_count
		dACC		21	gene ~ trait + SVs + Handedness + Delta.9.THC.Active + Tramadol + Amphetamines + Ketamine + personal_count
		BLA		25	gene ~ trait + SVs + Delta.9.THC.Active + Barbiturates + Anticholinergics..Antiparkinsonians + witness_count
		MeA		27	gene ~ trait + SVs + PTSD_PrimaryDx + Barbiturates + personal_count
	Cell-type	BLA	InN	54	gene ~ trait + SVs + Acetone + Cocaethylene + Hydrocodone + Ketamine + Anticholinergics..Antiparkinsonians + Antipsychotics + Hallucinogens + ECT + disaster_count
			ExN	43	gene ~ trait + SVs + Isopropanol + Cocaethylene + Past.Self.Mutilation + combat_occ_any
			Oligodendrocytes	43	gene ~ trait + SVs + X11.Hydroxy.Delta.9.THC.Active +

					Amphetamines + Barbiturates + Ketamine + disaster_count
			Endothelial	38	gene ~ trait + SVs + Handedness + Acetone + X11.Hydroxy.Delta.9.THC.Active + Barbiturates + Hallucinogens + childhood_count
			Astrocytes	49	gene ~ trait + SVs + Ketamine + Anticholinergics..Antiparkinsonians + Hallucinogens + ECT + witness_count + personal_count
			Microglia	28	gene ~ trait + SVs + Isopropanol + Cocaethylene + Barbiturates + Ketamine + Anticholinergics..Antiparkinsonians + Hallucinogens + ECT + witness_count + personal_count
		dACC	InN	38	gene ~ trait + SVs + BMI..calculated. + Isopropanol + Anti.Inflammatories + personal_count
			ExN	40	gene ~ trait + SVs + Cocaethylene + Benzoylecgonine_mg_L_blood + Anti.Inflammatories
			Oligodendrocytes	55	gene ~ trait + SVs + Cocaethylene + Benzoylecgonine_mg_L_blood + Barbiturates + Anticholinergics..Antiparkinsonians
			Endothelial	21	gene ~ trait + SVs + Cocaethylene + Nicotine_ng_mL + Amphetamines + Barbiturates + disaster_count + childhood_count
			Astrocytes	43	gene ~ trait + SVs + Cocaethylene + Benzoylecgonine_mg_L_blood + witness_count + combat_occ_count
			Microglia	12	gene ~ trait + SVs + Isopropanol + X11.Hydroxy.Delta.9.THC.Active + Cocaethylene + Benzoylecgonine_mg_L_blood + Hydrocodone +

					Barbiturates + Ketamine + Anticholinergics..Antiparkinsonians + witness_count
		DLPFC	InN	37	gene ~ trait + SVs + Cocaethylene + Hallucinogens + witness_count + personal_count
			ExN	36	gene ~ trait + SVs + Manner_of_death_Accident + Delta.9.THC.Active + Benzoylecgonine_mg_L_blood + Hallucinogens
			Oligodendrocytes	54	gene ~ trait + SVs + Handedness + Barbiturates + Ketamine + Anticholinergics..Antiparkinsonians + ECT + witness_count + personal_count + combat_occ_count
			Endothelial	17	gene ~ trait + SVs + BMI..calculated. + X11.Hydroxy.Delta.9.THC.Active + Cocaethylene + Tramadol + Barbiturates + personal_count
			Astrocytes	38	gene ~ trait + SVs
			Microglia	8	gene ~ trait + SVs + Handedness + Manner_of_death_Accident + Isopropanol + X11.Hydroxy.Delta.9.THC.Active + Benzoylecgonine_mg_L_blood + Anticholinergics..Antiparkinsonians + Anti.Inflammatories + Delusions combat_only_count
		MeA	InN	44	gene ~ trait + SVs + Acetone + Barbiturates + Ketamine + Hallucinogens + disaster_count + childhood_count
			ExN	50	gene ~ trait + SVs + Isopropanol + Ketamine + Anticholinergics..Antiparkinsonians + EA_UA_neglect_count
			Oligodendrocytes	46	gene ~ trait + SVs + GERD + Acetone + X11.Hydroxy.Delta.9.THC.Active +

					Benzoylecgonine_mg_L_blood + Barbiturates + Ketamine Anticholinergics..Antiparkinsonians witness_count
			Endothelial	41	gene ~ trait + SVs + Barbiturates + Hallucinogens + witness_count + personal_count
			Astrocytes	51	gene ~ trait + SVs + Cocaethylene + Benzoylecgonine_mg_L_blood + Hallucinogens + ECT + witness_count
			Microglia	24	gene ~ trait + SVs + Acetone + Cocaethylene + Nicotine_ng_mL + Barbiturates + Ketamine + Anticholinergics..Antiparkinsonians + Hallucinogens + non_combat_count
PRS	Cell-type	BLA	InN	49	gene ~ trait + SVs + Acetone + Cocaethylene + Hydrocodone + Ketamine + Anticholinergics..Antiparkinsonians + Antipsychotics + Last.PPD + ECT + disaster_count
			ExN	39	gene ~ trait + SVs + Race + Barbiturates + witness_count + combat_only_count
			Oligodendrocytes	37	gene ~ trait + SVs + Acetone + Amphetamines + Barbiturates + Ketamine + disaster_count + assault_count
			Endothelial	34	gene ~ trait + SVs + Acetone + X11.Hydroxy.Delta.9.THC.Active + Barbiturates + Hallucinogens + disaster_count + childhood_count
			Astrocytes	41	gene ~ trait + SVs + Acetone + Benzoylecgonine_mg_L_blood + Ketamine + Anticholinergics..Antiparkinsonians + Hallucinogens + ECT + witness_count

			Microglia	26	gene ~ trait + SVs + Acetone + Cocaethylene + Barbiturates + Ketamine + Anticholinergics..Antiparkinsonians + Hallucinogens + Age.Onset.Smoke + ECT + witness_count
		dACC	InN	33	gene ~ trait + SVs + Race+ Anti.Inflammatories + personal_count + age_num_first_trauma_infer
			ExN	34	gene ~ trait + SVs + Race + Acetone + Cocaethylene + Amphetamines + Age.Onset.Smoke + Past.Self.Mutilation
			Oligodendrocytes	45	gene ~ trait + SVs + Manner_of_death_Accident + Delta.9.THC.Active + Benzoylecgonine_mg_L_blood + Barbiturates + Anticholinergics..Antiparkinsonians
			Endothelial	19	gene ~ trait + SVs + Race + Acetone + Cocaethylene + Nicotine_ng_mL + Amphetamines + Barbiturates + witness_count + disaster_count
			Astrocytes	37	gene ~ trait + SVs + Race + Acetone + Cocaethylene + Amphetamines + Age.Onset.Smoke + witness_count
			Microglia	11	gene ~ trait + SVs + Handedness + Acetone + Ethanol_mg_dL + Benzoylecgonine_mg_L_blood + Acetaminophen + Ketamine + Anticholinergics..Antiparkinsonians + Hx.Military.Service + witness_any
		DLPFC	InN	34	gene ~ trait + SVs + Handedness + Benzoylecgonine_mg_L_blood + Age.Onset.Smoke + witness_any + combat_occ_any
			ExN	34	gene ~ trait + SVs + Race + Cocaethylene +

					Amphetamines + Barbiturates + personal_count + age_num_first_trauma_infer
			Oligodendrocytes	44	gene ~ trait + SVs + Race + Delta.9.THC.Active + Barbiturates + Ketamine + Anticholinergics..Antiparkinsonians + ECT + witness_count + personal_count
			Endothelial	16	gene ~ trait + SVs + X11.Hydroxy.Delta.9.THC.Active + Cocaethylene + Nicotine_ng_mL + Amphetamines + Barbiturates + Famhx.Suicide + disaster_count
			Astrocytes	34	gene ~ trait + SVs + Acetone + X11.Hydroxy.Delta.9.THC.Active + Cocaethylene + Amphetamines + combat_occ_count + childhood_count
			Microglia	8	gene ~ trait + SVs + Handedness + Ethanol + Acetone + Delta.9.Carboxy.THC.Inactive + Benzoylecgonine_mg_L_blood + Age.Onset.Smoke + Past.Self.Mutilation + ECT + Hx.Combat.Seen
		MeA	InN	42	gene ~ trait + SVs + Acetone + Barbiturates + Ketamine + Anticholinergics..Antiparkinsonians + Hallucinogens + ECT + disaster_count
			ExN	44	gene ~ trait + SVs + Ketamine + Anticholinergics..Antiparkinsonians + ECT + combat_occ_count
			Oligodendrocytes	41	gene ~ trait + SVs + X11.Hydroxy.Delta.9.THC.Active + Amphetamines + Barbiturates + Ketamine + Anticholinergics..Antiparkinsonians witness_count personal_count combat_occ_count

			Endothelial	36	gene ~ trait + SVs + Acetone + X11.Hydroxy.Delta.9.THC.Active + Barbiturates + Hallucinogens + disaster_count + childhood_count
			Astrocytes	42	gene ~ trait + SVs + plate + Cocaethylene + Amphetamines + childhood_count
			Microglia	22	gene ~ trait + SVs + Cocaethylene +Oxymorphone + Barbiturates + Ketamine + Anticholinergics..Antiparkinsonians + Hallucinogens + witness_count

Table S2: Additional DEGs in MeA and BLA OPCs.

Trait	Region	gene	Z	P	P_bonf
Chronic pain	BLA	YBX3	-5.1	7.69E-07	1.44E-02
		CD84	-5.81	2.29E-08	4.27E-04
		GADD45A	-5.68	4.48E-08	8.38E-04
		BAG3	-5.37	2.06E-07	3.86E-03
	MeA	DYNLL1	-4.9	1.88E-06	3.51E-02
		PDGFB	-5.51	9.82E-08	1.83E-03
		WDR83OS	-5.35	2.21E-07	4.14E-03
		ETV3	-5.23	3.87E-07	7.23E-03
		DUSP4	-5.09	7.54E-07	1.41E-02
		EGR2	-5.59	6.73E-08	1.26E-03
		COX16	-5.05	9.32E-07	1.74E-02
		TEC	4.86	2.29E-06	4.27E-02
		PROSC	-5.75	2.99E-08	5.60E-04
		UCN	5.26	3.36E-07	6.27E-03
		CRK	-4.94	1.52E-06	2.84E-02
		TRIB1	-5.09	7.56E-07	1.41E-02
		ZFP1	-5.32	2.53E-07	4.72E-03
		ZNF511	-5.04	9.87E-07	1.84E-02
		PRC1	4.95	1.46E-06	2.73E-02
		LBH	-4.96	1.45E-06	2.70E-02
		C1orf233	5.05	9.10E-07	1.70E-02
		GLUD1P3	4.95	1.51E-06	2.83E-02
Oxymorphone	BLA	MASP1	-4.87	2.24E-06	4.18E-02

		SPATA13	-4.83	2.64E-06	4.93E-02
		RP11.309I15.1	-4.83	2.64E-06	4.93E-02
	MeA	LBH	-5.01	1.19E-06	2.23E-02
Fentanyl	BLA	EPHA3	5.26	3.68E-07	6.88E-03
		LZTS1	5.45	1.51E-07	2.81E-03
		SREBF1	5.63	5.95E-08	1.11E-03
		CACNG5	5.23	4.37E-07	8.16E-03
		DBC1	5.07	8.94E-07	1.67E-02
		ITCH	4.9	1.98E-06	3.70E-02
		PGM1	5.74	3.41E-08	6.38E-04
		SMOX	4.95	1.58E-06	2.96E-02
		CDC23	4.98	1.38E-06	2.58E-02
		EIF5	-5.76	3.16E-08	5.90E-04
		KIAA0391	4.92	1.82E-06	3.41E-02
		TMEM87A	4.98	1.37E-06	2.56E-02
		CRYBA1	5.73	3.75E-08	7.00E-04
		TNFAIP1	5.65	5.44E-08	1.02E-03
		ARSB	5.46	1.42E-07	2.66E-03
		HES1	5.16	5.82E-07	1.09E-02
		PRDM2	-4.95	1.57E-06	2.94E-02
		APH1A	5.5	1.17E-07	2.18E-03
		FMO6P	5.1	8.00E-07	1.50E-02
		AKAP1	5.32	2.76E-07	5.15E-03
		SDC4	4.86	2.41E-06	4.51E-02
		ABCC10	-5.05	9.95E-07	1.86E-02
		SNAP25	-5.2	4.85E-07	9.07E-03
		MED10	-4.93	1.72E-06	3.21E-02
		RFK	-5.25	3.81E-07	7.11E-03
		ITM2C	4.91	1.89E-06	3.54E-02
		MRPS9	4.9	1.96E-06	3.65E-02
		CCL21	5.03	1.09E-06	2.03E-02
		GRHPR	4.91	1.89E-06	3.54E-02
		LRRC32	5.04	1.05E-06	1.97E-02
		TMBIM6	5.03	1.10E-06	2.05E-02
		C18orf8	-4.86	2.36E-06	4.40E-02
		GTDC2	5	1.26E-06	2.35E-02
		CDC123	4.9	1.98E-06	3.70E-02
		PFKM	5.65	5.37E-08	1.00E-03
		WNT7A	5.03	1.08E-06	2.01E-02

		SSR2	5.56	8.64E-08	1.61E-03
		CYBB	-5.01	1.22E-06	2.28E-02
		ILK	5.19	5.21E-07	9.74E-03
		PBX3	5.29	3.28E-07	6.14E-03
		ECI1	4.93	1.74E-06	3.26E-02
		SF1	-4.87	2.23E-06	4.17E-02
		CX3CR1	-4.94	1.65E-06	3.08E-02
		GPR34	-5.8	2.60E-08	4.86E-04
		LRRC20	5.1	7.97E-07	1.49E-02
		CES3	-5.02	1.14E-06	2.14E-02
		LINC00982	4.98	1.38E-06	2.59E-02
		CCDC89	-4.88	2.19E-06	4.09E-02
		C1orf194	-4.98	1.37E-06	2.56E-02
		P2RY13	-5.09	8.18E-07	1.53E-02
		SCN5A	6.53	5.25E-10	9.81E-06
		ZNF75D	4.85	2.50E-06	4.67E-02
		STMN3	5.25	3.94E-07	7.37E-03
		MPZL1	5.64	5.85E-08	1.09E-03
		DUSP27	-4.96	1.52E-06	2.84E-02
		SNORD115.2	5.17	5.76E-07	1.08E-02
		FAM71F2	-5.41	1.76E-07	3.29E-03
		RNVU1.18	-6.15	4.18E-09	7.82E-05
		MLLT11	-5.24	4.09E-07	7.64E-03
		AC009227.2	5.21	4.78E-07	8.93E-03
		RP11.180I22.2	5.2	5.00E-07	9.34E-03
		RP3.437I16.1	-5.57	8.22E-08	1.54E-03
		USP17L4	5.6	7.20E-08	1.35E-03
		MYLK.AS2	5.18	5.38E-07	1.00E-02
	MeA	NDUFAF7	-5.12	6.61E-07	1.23E-02
		NLE1	4.83	2.53E-06	4.74E-02
		POMGNT1	5.42	1.48E-07	2.77E-03
		RPLP0	4.95	1.43E-06	2.66E-02
		HIF1A	5.06	8.43E-07	1.57E-02
		PRPF6	5.14	5.99E-07	1.12E-02
		IDH3B	5.07	8.28E-07	1.55E-02
		NFAT5	-4.82	2.61E-06	4.87E-02
		MYEF2	-4.92	1.65E-06	3.09E-02
		MTCH2	5.12	6.51E-07	1.22E-02
		LDHB	6	7.51E-09	1.40E-04

		WASF1	-4.87	2.12E-06	3.95E-02
		SELK	4.95	1.42E-06	2.65E-02
		NKTR	-5.29	2.86E-07	5.35E-03
		PRDX1	5.82	2.01E-08	3.76E-04
		ALDH6A1	5.13	6.32E-07	1.18E-02
		PKN1	4.85	2.31E-06	4.31E-02
		MRPS7	5.24	3.63E-07	6.78E-03
		CASD1	-5.44	1.39E-07	2.60E-03
		EIF3G	5.53	8.71E-08	1.63E-03
		MGAT1	4.9	1.81E-06	3.39E-02
		RAMP1	5.39	1.74E-07	3.26E-03
		PEMT	5.48	1.13E-07	2.11E-03
		CDK4	5.06	8.48E-07	1.59E-02
		ITM2C	6.48	5.44E-10	1.02E-05
		SCRN1	5.28	3.08E-07	5.75E-03
		CREBZF	-5.31	2.61E-07	4.87E-03
		TRIM29	-5.19	4.62E-07	8.63E-03
		ETNK1	-4.93	1.58E-06	2.96E-02
		GALNT1	5.03	9.94E-07	1.86E-02
		MICU3	-5.25	3.50E-07	6.54E-03
		UBN2	-5.27	3.22E-07	6.02E-03
		FAM69B	5.5	9.92E-08	1.85E-03
		NDUFV1	5.33	2.30E-07	4.30E-03
		NSMCE1	5.25	3.46E-07	6.47E-03
		SNUPN	4.97	1.30E-06	2.42E-02
		PHC3	-6.79	9.83E-11	1.84E-06
		COA4	5.06	8.60E-07	1.61E-02
		PLCD1	5.65	4.75E-08	8.88E-04
		LIN54	-5.28	3.08E-07	5.76E-03
		TRIM33	-5.18	4.82E-07	9.01E-03
		SNORA38B	-5.23	3.92E-07	7.32E-03
		SNORA80	-5.83	1.89E-08	3.54E-04
		C9orf135	-4.85	2.32E-06	4.34E-02
		AC005594.3	-5.09	7.59E-07	1.42E-02
		SNORD15B	-4.88	1.96E-06	3.67E-02
		ACY1	5.27	3.22E-07	6.01E-03
Migraine	MeA	SPAG4	4.99	1.26E-06	2.36E-02
		CCDC91	-4.84	2.51E-06	4.69E-02
		ZDHHC12	4.83	2.62E-06	4.89E-02

Table S3: Pathways (gene sets) enriched for additional chronic pain cell type DEGs. Gene sets significantly ($p < 1.53 \times 10^{-6}$) enriched for cell type DEGs with minimum N gene overlap = 10. GO = Gene Ontology. No gene sets were found to be significantly enriched for dACC microglia, dACC oligodendrocytes, medial amygdala astrocytes, and medial amygdala endothelial cell type DEGs.

Region	Cell Type	Category	GeneSet	N_genes	N_overlap	p
MeA	Microglia	GO biological processes	GOBP_CYTOPLASMIC_TRANSLATION	125	14	5.16×10^{-14}
			GOBP_PEPTIDE_METABOLIC_PROCESS	744	20	4.12×10^{-08}
			GOBP_PEPTIDE_BIOSYNTHETIC_PROCESS	626	18	7.91×10^{-08}
			GOBP_AMIDE_BIOSYNTHETIC_PROCESS	752	18	1.17×10^{-06}
			GOBP_AMIDE_METABOLIC_PROCESS	1009	21	1.28×10^{-06}
		Chemical and Genetic pertubation	HSIAO_HOUSEKEEPING_GENES	332	14	2.46×10^{-08}
		GO cellular component	GOCC_CYTOSOLIC_RIBOSOME	67	13	2.57×10^{-16}
			GOCC_RIBOSOME	180	16	2.86×10^{-14}
			GOCC_RIBOSOMAL_SUBUNIT	149	15	3.04×10^{-14}
			GOCC_RIBONUCLEOPROTEIN_COMPLEX	668	19	3.89×10^{-08}
		GO molecular function	GOMF_STRUCTURAL_CONSTITUENT_OF_RIBOSOME	132	14	1.11×10^{-13}
			GOMF_STRUCTURAL_MOLECULE_ACTIVITY	591	18	3.31×10^{-08}
		Computational gene sets	MORF_TPT1	73	13	8.45×10^{-16}
			MODULE_83	266	19	5.08×10^{-15}
			MORF_ACTG1	105	13	1.16×10^{-13}
			GNF2_EIF3S6	93	12	6.46×10^{-13}
			MORF_NPM1	126	13	1.27×10^{-12}
			MODULE_114	285	17	2.92×10^{-12}
			MODULE_151	269	16	1.41×10^{-11}
			GNF2_FBL	117	11	2.06×10^{-10}
			MORF_NME2	126	11	4.60×10^{-10}
			MODULE_32	209	11	9.32×10^{-08}
		Cell type signature	TRAVAGLINI_LUNG_CD4_NAIVE_T_CELL	109	15	2.59×10^{-16}

			RUBENSTEIN_SKELETAL_MUSCLE_T_CELLS	142	15	$1.47x10^{-14}$
			BUSSLINGER_GASTRIC_PPP1R1B_POSITIVE_CELLS	92	13	$1.98x10^{-14}$
			TRAVAGLINI_LUNG_BRONCHIAL_VESSEL_1_CELL	196	16	$1.09x10^{-13}$
			HAY_BONE_MARROW_NAIVE_T_CELL	321	19	$1.53x10^{-13}$
			TRAVAGLINI_LUNG_CLUB_CELL	92	12	$5.65x10^{-13}$
			RUBENSTEIN_SKELETAL_MUSCLE_SATELLITE_CELLS	260	17	$6.65x10^{-13}$
			MURARO_PANCREAS_ACINAR_CELL	622	24	$7.86x10^{-13}$
			RUBENSTEIN_SKELETAL_MUSCLE_B_CELLS	128	13	$1.56x10^{-12}$
			BUSSLINGER_DUODENAL_TRANSIT_AMPLIFYING_CELLS	161	14	$1.76x10^{-12}$
			BUSSLINGER_DUODENAL_DIFFERENTIATING_STEM_CELLS	257	16	$7.08x10^{-12}$
			LAKE_ADULT_KIDNEY_C7_PROXIMAL_TUBULE_EPITHELIAL_CELLS_S3	106	11	$7.00x10^{-11}$
			BUSSLINGER_DUODENAL_IMMUNE_CELLS	807	24	$1.82x10^{-10}$
			LAKE_ADULT_KIDNEY_C10_THIN_ASCENDING_LIMB	305	15	$9.17x10^{-10}$
			LAKE_ADULT_KIDNEY_C12_THICK_ASCENDING_LIMB	341	15	$4.21x10^{-09}$
			BUSSLINGER_DUODENAL_STEM_CELLS	260	13	$1.08x10^{-08}$
			LAKE_ADULT_KIDNEY_C19_COLLECTING_DUCT_INTERCALATED_CELLS_TYPE_A_MEDULLA	283	13	$2.96x10^{-08}$
			RUBENSTEIN_SKELETAL_MUSCLE_PCV_E	188	11	$3.15x10^{-08}$

			NDOTHELIAL_CELLS			
			TRAVAGLINI_LUNG_CAPILLARY_INTERMEDIATE_2_CELL	353	14	5.29×10^{-08}
			TRAVAGLINI_LUNG_MESOTHELIAL_CELL	542	17	5.47×10^{-08}
			LAKE_ADULT_KIDNEY_C9_THIN_ASCENDING_LIMB	231	11	2.56×10^{-07}
		Canonical Pathways	REACTOME_EUKARYOTIC_TRANSLATION_ELONGATION	64	14	2.77×10^{-18}
			KEGG_RIBOSOME	60	13	5.42×10^{-17}
			WP_CYTOPLASMIC_RIBOSOMAL_PROTEINS	62	13	8.62×10^{-17}
			REACTOME_RESPONSE_OF_EIF2AK4_GCN2_TO_AMINO_ACID_DEFICIENCY	74	13	1.02×10^{-15}
			REACTOME_CELLULAR_RESPONSE_TO_STARVATION	122	15	1.47×10^{-15}
			REACTOME_NONSENSE_MEDIATED_DECAY_NMD	83	13	4.92×10^{-15}
			REACTOME_SRP_DEPENDENT_COTRANSLATIONAL_PROTEIN_TARGETING_TO_MEMBRANE	85	13	6.80×10^{-15}
			REACTOME_SELENOAMINO_ACID_METABOLISM	87	13	9.32×10^{-15}
			REACTOME_EUKARYOTIC_TRANSLATION_INITIATION	89	13	1.27×10^{-14}
			REACTOME_INFLUENZA_INFECTION	127	13	1.41×10^{-12}
			REACTOME_RRNA_PROCESSING	164	14	2.27×10^{-12}
			REACTOME_REGULATION_OF_EXPRESSION_OF_SLITS_AND_ROBOS	133	13	2.56×10^{-12}
			REACTOME_TRANSLATION	251	16	4.95×10^{-12}
			REACTOME_SIGNALING_BY_ROBO_RECEPTORS	177	13	9.74×10^{-11}

			REACTOME_DEVELOPMENTAL_BIOLOGY	833	22	$1.06x10^{-08}$
			REACTOME_NERVOUS_SYSTEM_DEVELOPMENT	522	17	$3.17x10^{-08}$
			REACTOME_METABOLISM_OF_AMINO_ACIDS_AND_DERIVATIVES	316	13	$1.08x10^{-07}$
			REACTOME_CELLULAR_RESPONSES_TO_STIMULI	688	18	$3.22x10^{-07}$
	Oligodendrocytes	Chemical and Genetic pertubation	BLALOCK_ALZHEIMERS_DISEASE_DN	1171	12	$3.46x10^{-08}$
		GO cellular component	GOCC_NEURON_PROJECTION	1241	11	$7.10x10^{-07}$
		Cell type signature	MURARO_PANCREAS_BETA_CELL	875	11	$2.02x10^{-08}$

Table S4: Several genes are differentially expressed in medial amygdala cell types in both chronic pain and migraine. P_bonf = Bonferroni-corrected p value, Z = DEG regression beta value divided by DEG regression standard error.

		**Chronic pain**			**Migraine**		
Cell	Gene	Z	P	P_bonf	Z	P	P_bonf
Endothelial	GLUD1P3	6.98	3.48E-11	6.50E-07	4.94	1.51E-06	2.83E-02
	KDM4B	5.95	1.06E-08	1.97E-04	4.91	1.69E-06	3.15E-02
	LIFR	-4.9	1.86E-06	3.47E-02	-5.92	1.14E-08	2.13E-04
	NRBF2	-4.95	1.49E-06	2.79E-02	-6.09	4.48E-09	8.36E-05
	TNFRSF6B	5.18	4.92E-07	9.19E-03	4.89	1.85E-06	3.47E-02
Microglia	CEBPD	5.21	4.14E-07	7.73E-03	5.19	4.52E-07	8.45E-03
	MBNL1	5.18	4.86E-07	9.07E-03	5.15	5.61E-07	1.05E-02
	MRPL39	4.89	1.87E-06	3.49E-02	5.19	4.63E-07	8.65E-03
	RBM22	-5.1	6.96E-07	1.30E-02	4.98	1.25E-06	2.34E-02
	RPL24	-5.21	4.19E-07	7.83E-03	4.83	2.53E-06	4.74E-02
	RPL27	-5.52	9.31E-08	1.74E-03	4.9	1.80E-06	3.37E-02
	RRBP1	5.04	9.28E-07	1.73E-02	6.31	1.40E-09	2.61E-05

Table S5: Overlap in migraine and chronic pain DEGs is primarily driven by medial amygdala endothelial cells. P = hypergeometric test p value.

**Region**	**Cell type**	**N DEG overlap**	**p**
dACC	Astrocytes	0	1
	Endothelial	0	1
	ExN	0	1
	InN	0	1
	Microglia	0	1
	Oligodendrocytes	0	1
DLPFC	Astrocytes	0	1
	Endothelial	0	1
	ExN	0	1
	InN	0	1
	Microglia	0	1
	Oligodendrocytes	0	1
BLA	Astrocytes	0	1
	Endothelial	0	1
	ExN	0	1
	InN	0	1
	Microglia	0	1
	Oligodendrocytes	0	1
MeA	Astrocytes	0	1
	Endothelial	5	0.022
	ExN	0	1
	InN	0	1
	Microglia	7	0.16
	Oligodendrocytes	0	1

Table S6: Several genes are differentially expressed in amygdala cell types in both chronic pain and lifetime oxymorphone use. P_bonf = Bonferroni-corrected p value, Z = DEG regression beta value divided by DEG regression standard error.

			**Chronic pain**			**Oxymorphone**		
Region	Cell	Gene	Z	P	P_bonf	Z	P	P_bonf
BLA	Endothelial	GADD45B	4.87	1.95E-06	0.03637	6.3	1.51E-09	2.82E-05
		YBX3	4.81	2.63E-06	0.049103	6.15	3.42E-09	6.40E-05
	Microglia	CCL2	5.7	3.49E-08	0.000652	4.92	1.76E-06	3.30E-02
		RASSF10	-5.36	2.02E-07	0.003779	-4.98	1.38E-06	2.59E-02
		SERPINA3	5.21	4.06E-07	0.007581	4.95	1.55E-06	2.90E-02
MeA	Endothelial	FAM20C	5.24	3.77E-07	0.007041	4.95	1.44E-06	2.68E-02
		HILPDA	5.26	3.35E-07	0.006261	4.87	2.09E-06	3.91E-02
		KRBA1	5.35	2.22E-07	0.004151	5.15	5.65E-07	1.06E-02
		LOXL2	4.83	2.57E-06	0.047982	4.95	1.42E-06	2.66E-02
	Microglia	FZD8	-5.55	7.80E-08	0.001457	-4.94	1.52E-06	2.84E-02
		RPL27	-5.52	9.31E-08	0.001739	-4.99	1.17E-06	2.19E-02
		RPL31	-6	7.44E-09	0.000139	-4.91	1.75E-06	3.27E-02
		RPS21	-5.68	3.98E-08	0.000744	-5.49	1.05E-07	1.97E-03

Table S7: Overlap in lifetime oxymorphone use and chronic pain DEGs is primarily driven by amygdala endothelial cells. P = hypergeometric test p value.

Region	Region and cell type	N DEG overlap	p
dACC	Astrocytes	0	1
	Endothelial	0	1
	ExN	0	1
	InN	0	1
	Microglia	0	1
	Oligodendrocytes	0	1
DLPFC	Astrocytes	0	1
	Endothelial	0	1
	ExN	0	1
	InN	0	1
	Microglia	0	1
	Oligodendrocytes	0	1
BLA	Astrocytes	0	1
	Endothelial	2	9×10^{-04}
	ExN	0	1
	InN	0	1
	Microglia	3	8×10^{-03}
	Oligodendrocytes	0	1
MeA	Astrocytes	0	1
	Endothelial	4	2×10^{-04}
	ExN	0	1
	InN	0	1
	Microglia	4	7×10^{-03}
	Oligodendrocytes	0	1

Table S8: Genes differentially expressed in lifetime fentanyl use. pBonferroni = Bonferroni-corrected p value, Z = DEG regression beta value divided by DEG regression standard error. ExN = excitatory neuron, InN = inhibitory neuron.

**Region**	**cell**	**Gene**	**p**	$p_{Bonferroni}$	**Z**
dACC	Endothelial	PIK3R5	$1.07x10^{-06}$	0.0200	-5
	ExN	PIK3R5	$4.21x10^{-07}$	$7.88x10^{-03}$	-5.195
	InN	PIK3R5	$1.97x10^{-06}$	0.0367	-4.876
BLA	Endothelial	EXTL3	$1.53x10^{-06}$	0.0286	4.935
		PHLPP2	$1.87x10^{-06}$	0.0350	4.892
		GSK3B	$2.41x10^{-06}$	0.0451	4.836
		NUP50	$9.66x10^{-07}$	0.0181	5.033
		MS4A4A	$8.41x10^{-07}$	0.0157	-5.063
		ASTN1	$1.66x10^{-06}$	0.0311	4.917
		GNE	$4.83x10^{-07}$	$9.03 x10^{-03}$	5.179
		HPGDS	$8.35x10^{-07}$	0.0156	-5.064
		TCEAL7	$2.32x10^{-07}$	$4.32 x10^{-03}$	-5.331
		CDRT15	$6.43x10^{-08}$	$1.20 x10^{-03}$	-5.588
	ExN	ZNF806	$5.03x10^{-07}$	$9.39 x10^{-03}$	-5.164
		YWHAQ	$8.70x10^{-07}$	0.0163	-5.049
		AKAP17A	$1.68x10^{-07}$	$3.13 x10^{-03}$	-5.389
MeA	Astrocytes	LINC00863	$5.16x10^{-07}$	$9.64 x10^{-03}$	-5.213

References

1. Newman, A. M. *et al.* Determining cell type abundance and expression from bulk tissues with digital cytometry. *Nat. Biotechnol.* **37**, 773–782 (2019).
2. Barrett, T. *et al.* NCBI GEO: archive for functional genomics data sets—update. *Nucleic Acids Res.* **41**, D991–D995 (2013).
3. Yu, B. *et al.* Molecular and cellular evolution of the amygdala across species analyzed by single-nucleus transcriptome profiling. *Cell Discov.* **9**, 1–22 (2023).
4. Satija, R., Farrell, J. A., Gennert, D., Schier, A. F. & Regev, A. Spatial reconstruction of single-cell gene expression data. *Nat. Biotechnol.* **33**, 495–502 (2015).
5. Smedley, D. *et al.* BioMart – biological queries made easy. *BMC Genomics* **10**, 22 (2009).
6. Liberzon, A. *et al.* The Molecular Signatures Database (MSigDB) hallmark gene set collection. *Cell Syst.* **1**, 417–425 (2015).
